# Post mortem findings in sows and gilts euthanised or found dead in a large Swedish herd

**DOI:** 10.1186/1751-0147-50-25

**Published:** 2008-07-01

**Authors:** Linda Engblom, Lena Eliasson-Selling, Nils Lundeheim, Katinka Belák, Kjell Andersson, Anne-Marie Dalin

**Affiliations:** 1 Department of Animal Breeding and Genetics, Swedish University of Agricultural Sciences, P.O. Box 7023, SE-750 07, Uppsala, Sweden; 2 Swedish Animal Health Service, Kungsängens gård, SE-753 23, Uppsala, Sweden; 3 National Veterinary Institute, SE-751 89, Uppsala, Sweden; 4 Department of Clinical Sciences, Division of Reproduction, Swedish University of Agricultural Sciences, P.O. Box 7054, SE-750 07, Uppsala, Sweden

## Abstract

**Background:**

The aim of this study was to get information on post mortem diagnoses of sows found dead or euthanised and to understand the diagnoses aetiology (causative background). Moreover, the study was to evaluate the association between the clinical symptoms observed on farm and post mortem findings.

**Methods:**

A large Swedish herd was studied from January to September 2006. During the 32-week period 3.9% of the removed sows and gilts (old enough to be mated) were found dead, 12.0% were euthanised and the rest were sent to slaughter. Of 32 sows/gilts found dead 17 (53%) were post mortem examined, and of 98 sows euthanised 79 (81%) were examined. The 96 examined carcasses were after 70 sows and 26 gilts. The findings at examination were together with data from the herd monitoring program PigWin Sugg the base for the descriptive statistics presented.

**Results:**

The average parity number at removal was 2.8 for those found dead and 2.1 for those euthanised. The highest number euthanised and found dead was in parity 0 (gilts). The main proportion of post mortem examinations was made on sows being in the period = 28 d of gestation at death (37.5%), followed by weaning to next service period (24.0%). Arthritis, with an incidence of 36.4% was the most common main finding of pathological-anatomical diagnosis (PAD). Of sows/gilts found dead were circulatory/cardiac failure (23.5%) and trauma related injuries (23.5%) most common PAD. The most commonly observed clinical symptom and reason for euthanasia of the sows/gilts was lameness. Notably, in 43% of the cases with PAD arthritis, the clinical symptoms suggested it being a fracture. Further one or more abscesses (38.5%) and teeth injuries (31.0%) were common findings when also incidental findings were included.

**Conclusion:**

This post mortem study based on carcasses from sows/gilts found dead or euthanised showed that arthritis was a significant problem in the studied herd and that post mortem examination was important to get proper diagnosis.

## Background

Sow mortality includes sows found dead. However, sows euthanised on farm due to trauma or disease are generally also included in studies on mortality. Both of these two kinds of unplanned removal lead to urgent need for replacement gilts, loss of income from slaughter, and an extra cost for destruction of the carcasses. Besides the loss in production, there is a risk that the sows are suffering from pain during their last days alive. Annual mortality rates reported previously, mainly including sows found dead, varied from 3.4% to 6.9% of sows in production [1-3]. Risk factors for sow mortality have been identified. Higher mortality was reported during summer months [2,4,5], both during the days before expected farrowing and the days just after farrowing [4,6,7].

In two recent studies, from Denmark and USA, the proportion euthanised and found dead were of equal magnitude [8,9] whereas a Swedish study reported more than two times as many euthanised sows as found dead [10]. The primary findings at post mortem examination of sows that die in the herd vary between studies. Heart failure was the most common finding in two Canadian studies [4,11] whereas locomotory related findings was reported as most common in others [9,12].

This study is a part of a larger investigation of sow removal in Swedish commercial herds. Removal pattern [10] and factors affecting length of productive life [13] have been investigated. The aim of this study was to get information on post mortem diagnosis of sows euthanised or found dead, i.e. to obtain the proper diagnoses. Moreover, the study was to evaluate the association between the clinical symptoms observed on farm and the post mortem findings.

## Methods

This study was based on material collected from a sow pool with 2200 crossbred Landrace × Yorkshire sows in the south central part of Sweden. The sow pool had a central unit supplying 13 satellite units with pregnant sows within a leasing system. In the central unit the newly weaned sows were housed in groups of 50 on deep straw bedding for one week. During this period oestrus was checked twice daily starting three days after weaning and artificial insemination was used. During the first eight weeks of pregnancy the sows were kept in smaller groups (9–15 sows per pen) on concrete/partially slatted floor with access to straw. Pregnancy check was performed twice with ultrasound scan, at 4 and 8 weeks. After the second check, pregnant sows were moved to large pens (50 sows per pen) with deep straw or peat bedding.

Three weeks before expected farrowing, sows were transported to satellite units where they on arrival were housed in groups on deep straw bedding. A couple of days before expected farrowing, the sows were moved into the individual farrowing pens where they stayed with their litter during the lactation period (approximately 5 weeks). After weaning, sows were transported back to the central unit either to be mated for the next reproductive cycle or to be sent for slaughter. All satellite units were located within a one-hour drive from the central unit and sows were transported in a two-storey ventilated lorry.

At the site of the central unit there was also a multiplying unit with 250 purebred Yorkshire and Landrace sows producing crossbred gilts for the sow pool. The routines in this multiplying unit were the same as in the sow pool itself as described above, except that these sows stayed at the same location throughout the reproductive cycle. Growing gilts were housed in pens with concrete/partially slatted floors. The feed in the sow pool was based on home produced grain (mainly barley) supplemented with commercial concentrate. In the central unit, the multiplying unit and in ten of the satellite units the feed was given in liquid form, while in the remaining four satellite units dry feed was given.

### Post mortem examination

From January 22 to September 4, 2006, a total of 130 sows and gilts (old enough to be mated; parity 0) were euthanised or found dead in the central unit, satellites units or in the multiplying unit. At euthanasia a captive bolt pistol was used and blood was drained. For sows/gilts euthanised or found dead, a form was to be filled in by the herd staff. The form included the sow's identity, parity number, date, stage of the reproductive cycle, observed clinical symptoms (more than one symptom could be reported) and if any medical treatment had been administered recently. Sows/gilts carcasses were transported to the National Veterinary Institute (SVA) in Uppsala where post mortem examination was performed according to standard procedure. No post mortem examination was performed on Saturdays and Sundays so sows/gilts euthanised or found dead from Friday afternoon to Saturday lunch were not examined. In total 34 carcasses were not examined, including those the herdsmen due to logistical matters were unable to transport to SVA, resulting in 96 examined carcasses.

The condition of the 96 carcasses was evaluated. Carcasses were cut open and all inner organs were removed and examined. When necessary, bacteriological and histological samples were taken for further analysis. Moreover weight, backfat depth and teeth status (missing, broken or severely worn) were included and this information was recorded on 95, 59 and 85 carcasses, respectively. Furthermore, body condition score was recorded on 82 of the carcasses using a 5-grade scale (1 = cachectic; 3 = normal; 5 = obese).

### Data

Besides records on sows from post mortem examination, production data for each individual sow/gilt was collected from the PC-based herd monitoring program PigWin Sugg (Quality Genetics HB, Hörby) used at all units of the herd. Descriptive statistics on the data was performed using the SAS program, version 9 (SAS Institute Inc, Cary, NC, USA). Incidence rate was calculated to estimate the number of sows/gilts that were euthanised or found dead per day in the different reproductive stages. These figures were used to calculate the incidence rate ratio (IR). To test the distribution of sows/gilts removed (euthanised or found dead) over month and over parity number, in relation to number of days and parity number distribution in the herd respectively, X^2- ^tests were performed. In the X^2- ^tests of distribution of euthanised sows/gilts the months January and February were grouped together as were August and September. In the X^2 ^tests of sows found dead the months were grouped as follows: January-March, April-June and July-September. Moreover parity numbers were in the X^2 ^tests of sows found dead grouped into parity 0, parity 1–3 and parity 4–7. The primary pathological-anatomical diagnoses (PAD) from post mortem examinations were grouped into PAD categories (Table 1) and assigned trauma or disease based on the aetiology.

**Table 1:** Pathological-anatomical diagnosis (PAD) category and primary PAD from post mortem examination

Aetiology	PAD category	Primary PAD
Trauma	- fracture	bone fractures
	- miscellaneous	internal bleedings, haematoma, intestinal ruptures, dislocation of vertebrae
	
Disease	- arthritis	arthritis
	- osteochondrosis	osteochondrosis, epiphyseolysis
	- abscess in spinal cord	abscess in spinal cord
	- circulatory/cardiac failure	circulatory/cardiac failure
	- heart/lung inflammatory reaction	pneumonia, abscesses in lung, endocarditis
	- gastrointestinal inflammatory reaction	gastritis/ulcers and enteritis
	- miscellaneous	otitis media, mastitis, malignant lymphoma, spinal axon degeneration, malformed hoof, hepatitis (caused by Ascaris suis), abscess
	
Unknown		4 due to autolysis, 1 negative section

Data were collected from January 22 to September 4 2006 from a large Swedish herd.

## Results

During the 32-week period, 816 sows (n = 709) and gilts (n = 107) were removed from the sow pool including the multiplying unit. Average parity number at removal was 4.1. Of the removed sows/gilts, 3.9% were found dead, 12.0% were euthanised and the rest were sent to slaughter. This corresponds to an annual removal rate of sows at 46.7% of sows in production.

Of 32 sows/gilts found dead 17 (53%) were post mortem examined, and of 98 sows euthanised 79 (81%) were examined (Table 2). The distribution of the 96 sows/gilts examined was evaluated against all 130 sows/gilts found dead or euthanised during the period studied regarding parity number, season, reproductive stage and observed symptoms (data not shown). The 96 sows/gilts post mortem examined were considered to be a representative sample of the 130 (results not shown).

**Table 2:** Descriptive statistics of sows/gilts found dead or euthanized

	Found dead	Euthanised
Total number	32	98
Proportion of all removed during the study	3.9%	12.0%
Proportion of sows in production (SIP)	2.1%	6.5%
Post mortem examined	17	79
Proportion post mortem examined	53%	81%
Parity number, mean (min-max)	2.8 (0–7)	2.1 (0–7)
Weight (kg), mean (min-max)	220 (116–322)	176 (104–240)
Backfat depth (mm), mean (min-max)	19.8 (10–28)	15.7 (4–35)
Body score *, mean (min-max)	2.6 (1–3)	2.8 (1–4)
Medically treated a short time before death	29%	58%

* body score using a 5-grade scale (1 = cachectic; 3 = normal; 5 = obese).

Data were collected from January 22 to September 4 2006 from a large Swedish herd.

The proportion of sows/gilts being medically treated a short time before death was higher among animals being euthanised (58%) than for animals found dead (29%), see Table 2.

**Figure 1:**
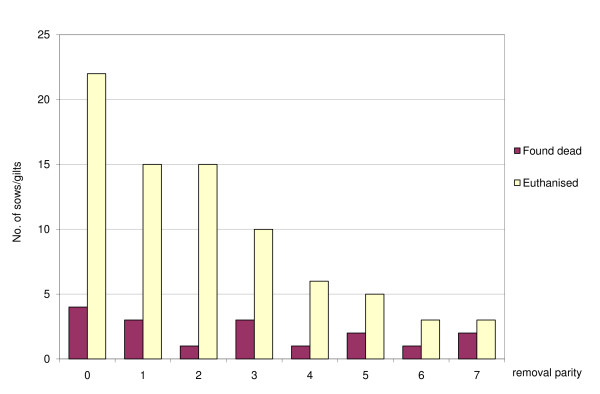
**Distribution of removal parity for 96 post mortem examined sows/gilts found dead or euthanised**. Data were collected from January 22 to September 4 2006 from a large Swedish herd.

**Figure 2:**
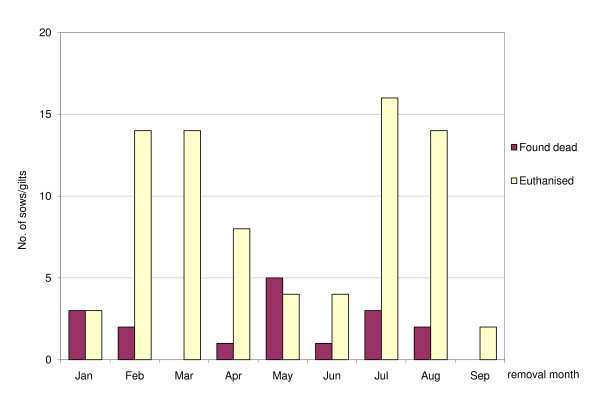
**Distribution of removal month for 96 post mortem examined sows/gilts found dead or euthanised**. Data were collected from January 22 to September 4 2006 from a large Swedish herd.

Of the 96 post mortem examined carcasses, 70 were from sows and 26 from gilts. The average parity number at removal for the sows/gilts was 2.8 for those found dead and 2.1 for those euthanised (Table 2). The highest number of euthanised and found dead were found in parity 0 (gilts), see Figure 1. The distribution of sows found dead did not deviate from the parity number distribution in the herd (p < 0.7), whereas the distribution of sows/gilts euthanised tended to deviate from the parity number distribution in the herd (p < 0.08). Examined sows had a higher proportion euthanized in parity 1 and 2 but a lower proportion in parity 7 than expected. Most sows/gilts were found dead in May whereas the number of sows/gilts euthanised was high in February, March, July and August (Figure 2). The distribution of sows found dead did not deviate from the expected even distribution when grouped into 3-month periods (p < 1.0), whereas the distribution of sows/gilts euthanised deviated from the expected even distribution during the period (p < 0.03).

Table 3 shows the distribution of sows/gilts euthanised or found dead according to reproductive stage. The main proportion of the examined sows/gilts were in the period = 28 d of gestation at death (37.5%), followed by sows in the weaning to next service period (24.0%), Table 3. In all the reproductive stages shown in Table 3, a higher proportion of sows/gilts was euthanised than found dead. The number of animals found dead was highest among sows/gilts in the period = 28 d of gestation (9 animals). Of these, 4 died after 100 d of gestation. The incidence rate ratio (IR) for sows euthanised or found dead during the two gestation periods and during the lactating period was approximately 1. However, the IR during weaning to service period was about 9 times higher than in the two gestation periods and the lactation period.

**Table 3:** Descriptive statistics by reproductive stage for 96 post mortem examined sows/gilts found dead or euthanised

Reproductive stage	Type of death	Most common PAD^1 ^category
Unmated gilts (n = 14) 14.6%	Found dead (n = 3) 21%	Circulatory/cardiac failure (n = 3)
	Euthanasia (n = 11) 79%	Arthritis (n = 8)
Weaning to service (n = 23) 24.0%	Found dead (n = 1) 4%	Heart/lung inflammatory reaction
	Euthanasia (n = 22) 96%	Arthritis (n = 8)
		Fracture (n = 6)
		Osteochondrosis (n = 4)
< 28 d gestation (n = 10) 10.4%	Found dead (n = 0)	-
	Euthanasia (n = 10) 100%	Arthritis (n = 4)
		Fracture (n = 2)
≥28 d gestation^2 ^ (n = 36) 37.5%	Found dead (n = 9) 25%	Not established (n = 3)
		Heart/lung inflammatory reaction (n = 2)
		Trauma miscellaneous (n = 2)
	Euthanasia (n = 27) 75%	Arthritis (n = 10)
		Osteochondrosis (n = 4)
		Abscess in spinal cord (n = 4)
With litter (n = 13) 13.5%	Found dead (n = 4) 31%	Gastrointestinal inflammat. react. (n = 2)
		Trauma miscellaneous (n = 2)
	Euthanasia (n = 9) 69%	Arthritis (n = 5)
		Osteochondrosis (n = 3)

^1 ^PAD = pathological-anatomical diagnosis

^2 ^includes 12 inseminated gilts

Data were collected from January 22 to September 4 2006 from a large Swedish herd.

The PAD categories are shown in Table 4. Most sows/gilts had disease related diagnoses. Arthritis was the single most common finding (36.4%), found in euthanised animals with average parity number 1.9 (Table 4). Almost all arthritis cases (34 out of 35) were chronic, purulent type and commonly found in elbow, stifle and/or shoulder. In 16 cases the arthritis was polyarthritic and in 14 cases polyarthritic and periarthritis. In 5 cases of purulent arthritis, samples were taken for bacteriological culture and 3 were found negative and 2 positive (Streptococcus equisimilis and Arcanobacterium pyogenes).

**Table 4:** Descriptive statistics by primary diagnosis category for 96 post mortem examined sows/gilts

				Type of death*		Average			
					
Aetiology	PAD^1 ^category	n	Prop (%)	1	2	Parity nr^2^	Weight (kg)	Back- fat (mm)	Body score^3 ^ (1–5)
Trauma n = 15 (15.6%)	- fracture	10	10.4	0	10	2.1	186	13.7	2.9
	- miscellaneous	5	5.2	4	1	1.6	210	19.3	2.8
	
Disease n = 76 (79.2%)	- arthritis^4^	35	36.4	0	35	1.9	164	15.6	2.6
	- osteochondrosis	13	13.5	0	13	2.1	185	10.9	2.9
	- abscess in spinal cord	7	7.3	0	7	3.7	210	19.2	3.0
	- circulatory/cardiac failure	4	4.2	4	0	0.8	157	25.5	3.0
	- heart/lung infl. react.	3	3.1	3	0	3.3	207	16.7	2.0
	- gastrointest. infl. react.	5	5.2	2	3	2.6	200	21.5	2.8
	- miscellaneous	9	8.3	1	8	1.6	180	13.3	3.0
	
Unknown n = 5 (5.2%)		5	5.2	3	2	3.6	245	23.8	3.0

*Type of death 1 = found dead, 2 = euthanised

^1 ^PAD = pathological-anatomical finding,

^2 ^includes also gilts (parity 0)

^3 ^body score using a 5-grade scale (1 = cachectic; 3 = normal; 5 = obese).

^4 ^includes also 1 case of arthritis caused by trauma

Data were collected from January 22 to September 4 2006 from a large Swedish herd.

The incidence of osteochondrosis was 13.5%, found in animals with average parity number 2.1 (Table 4). All animals with PAD osteochondrosis were euthanised. Among sows/gilts found dead, circulatory/cardiac failure and trauma related injuries were common main findings. Circulatory/cardiac failure was characterized pathoanatomically by an acute pulmonary oedema and passive congestion of lungs and liver with absence of gross and microscopic findings suggestive of other diseases. Three of the four animals that died due to circulatory/cardiac failure were gilts. The highest average parity number was found among animals with the PAD abscess in spinal cord (3.7), see Table 4.

The herd staff recorded the clinical symptoms before death of the sows/gilts that were euthanised or found dead. Table 5 shows the association between the clinical observations and the PAD categories. More than one clinical symptom could be reported per individual, but among sows found dead, 71% were found dead without any previous symptom. Lameness was the most common observed symptom for the sows/gilts with the primary findings fracture, arthritis and osteochondrosis. Notably, according to the clinical observations, fracture was expected to be found in 43% of the sows with the primary post mortem finding arthritis.

**Table 5:** Most common observed clinical symptoms* associated with PAD^1 ^categories in 96 post mortem examined sows/gilts

Aetiology	PAD^1 ^category	Most common clinical symptoms before death
Trauma	- fracture	80% lameness, 60% fracture
	- miscellaneous	80% none
	
	- arthritis	77% lameness, 43% fracture, 37% swollen joint
	- osteochondrosis	62% lameness, 38% sitting, 38% weight loss
	- abscess in spinal cord	86% sitting, 29% lameness, 29% spasm/lack of coordination
	
Disease		
	- circulatory/cardiac failure	75% none
	- heart/lunginfl. react.	67% sitting, 67% weight loss
	- gastroint. infl. react.	40% sitting, 40% inappetence
	- miscellaneous	44% sitting, 33% inappetence, 33% thin
	
Unknown		40% sitting, 40% none

* more than one symptom could be reported

^1 ^PAD = pathological-anatomical diagnosis

Data were collected from January 22 to September 4 2006 from a large Swedish herd.

Sows/gilts that got the primary finding trauma miscellaneous, circulatory/cardiac failure or unknown were in most cases found dead without any previous observed symptoms. Sitting (i.e. unable to stand up with the hind legs) was one commonly observed symptom for sows with PAD category abscess in spinal cord or heart/lung inflammatory reaction.

The pathological-anatomical findings, including most of the incidental findings, from the 96 examinations are listed in Table 6. The most common finding was arthritis (44.8%), followed by one or more abscesses (38.5%) and teeth injuries (31.0%). Teeth injuries were in approximately 50% of the cases found in the incicives.

**Table 6:** Descriptive statistics on pathological-anatomical findings, including most incidental finding, in 96 post mortem examined sows/gilts

	Found dead (n = 17)	Euthanised (n = 79)	Total (n = 96)	
	
	No.	No.	No.	%
Arthritis	2	41	43	44.8
Abscess, at least one	3	34	37	38.5
Teeth injuries	6/15	21/72	27/85	31.0
Osteochondrosis/epiphysiolysis	0	21	21	21.9
Kidney/urinary bladder failure	4	12	16	16.7
Pneumonia (App, SEP)	1	11	12	12.5
Mastitis	4	7	11	11.5
Fracture	0	10	10	10.4
Gastritis and/or ulceration	1	9	10	10.4
Heart disorders	5	5	10	10.4
Claw disorders	2	6	8	8.3
Abscess in spinal cord	0	7	7	7.3
Liver disorders	2	2	4	4.2
Reproductive organs	0	3	3	3.1
Spleen disorders	1	1	2	2.1

Data were collected from January 22 to September 4 2006 from a large Swedish herd.

## Discussion

This study was based on material from one herd, but the proportion of sows/gilts found dead and euthanised agrees with the findings in a larger study based on 21 Swedish piglet producing herds [10]. The high proportion of euthanised sows/gilts in both studies (12% and 10.5%) is partly due to the animal welfare legislation in Sweden, which states that only sows and gilts in normal body condition and without lameness are allowed to be transported to slaughter.

The proportion euthanised sows of all removed sows/gilts was in the present study about the same as that in a Danish study (10%) [8]. However the proportion found dead was markedly lower (4%) in the present study than in the Danish study (11%). Moreover the proportion of sows/gilts found dead of sows in production (2.1%) was lower than in previous studies from other countries [1,3,5]. The lower mortality found in the present study could be due to differences in housing, management system and climate. The high number of animals euthanised or found dead in low parity numbers corresponds to the age structure with many young animals in the herd. Other studies have shown higher mortality risk in higher parity numbers [5,13].

Most of the examined sows/gilts (79 of 96) in the present study were euthanised. Other studies of post mortem examined sows have had more equal proportion found dead and euthanised [8,9]. The variation in proportion found dead versus euthanised also influences the most common PAD at post mortem examination. The most common reason for euthanasia was lameness which agrees with a previous report [14]. It has earlier been reported that concrete/slatted floor increases the incidence of lameness [15] and in the present study sows/gilts were during a part of their gestation housed on concrete floor in groups.

In the present study the most common PAD was arthritis causing lameness resulting in the high proportion of euthanised sows/gilts. The high proportion of arthritis agrees with other studies on sow mortality [8,9,16]. In the present study, all arthritis cases except one were purulent and of chronic character. In nearly half of the cases more than one joint was affected, i.e. polyarthritic. The cause of these infectious arthritis cases was likely an initial trauma, e.g. due to group-housing (mounting during oestrus or rank order fighting) and secondary infection with bacterial infection with bacterial spread to multiple joints. The exact aetiology of these arthritis cases is questionable because of the lesions' chronic character. In the present study, the 35 arthritis cases were from relatively young animals (average parity number 1.9) and 10 were from gilts, which is remarkable. The background for this needs to be further studied.

Another common cause of lameness or leg weakness is osteochondrosis [17]. In present study osteochondrosis was the second most common primary diagnosis (all euthanised) and was not only found among young sows but also in older sows. Heredity plays a significant role for the development of osteochondrosis [17,18]. Since 1988, osteochondrosis has been included in the Swedish breeding evaluation. The finding that osteochondrosis was common among the post mortem examined sows/gilts, i.e. is still a significant problem, indicates the importance of increasing the selection pressure on constitution and osteochondrois. Besides genetic selection, feeding intensity [19], growth rate [18] and mechanical stress influence the incidence of osteochondrosis. In the present study, fracture was a common primary diagnosis in sows euthanised in the period weaning to service. Since the herd after weaning kept the sows loose-housed in large pens on deep straw bedding in the breeding area, the fractures probably occurred when sows in oestrus mounted each other or fought to establish rank order.

The proportion of animals with circulatory/cardiac failures in the present study was lower than in two Canadian studies [2,4] reporting circulatory failure as the most common primary finding for sow mortality. In the present study, circulatory/cardiac failure accounted for death in approximately 25% of the found dead post mortem examined sows, and among those, 3 out of 4 were non-mated gilts (parity 0). The probable cause of circulatory/cardiac failure in the present study may have been stress due to grouping. In the Canadian studies the average age for sows diagnosed with circulatory failure was higher, 2.3 years [2] and 4.1 parities [4], respectively. In one of these studies [2] circulatory failure was associated with heat stress. The lack of circulatory/cardiac failures due to heat stress in older sows in the present study may be due to a cold Swedish summer in 2006. The incidence of sows with gastric ulcers found in the present study was low compared with other studies [8,9,20]. In the present study, sows had access to straw and straw has been reported to decrease the risk of mortality due to gastrointestinal disorders [12].

Abscess in the spinal cord accounted for 7% of the PAD. In other studies this diagnosis has not been reported. An association between tail biting and carcass abscesses have been reported [21]. Although tail status of the carcasses was not recorded, clinical observations do not indicate problem with tail biting in the herd. In the present study no sow had a primary finding related to urinary or reproductive organs, which differs from other studies [9,12,20]. This may be due to the advantages of the loose housing system since urinary tract disease has been reported to be more common in sows tethered or kept in individual stalls [22] due to ascending infections. The lower incidence of reproductive problems in the present study compared with other studies may be due to the same reason [22]. The factors behind might be that loose-housed sows get better physical condition than individual stalled/tethered sows, which may result in fewer problems at farrowing, e.g. secondary uterine inertia (laber).

In the present study, the herd staff recorded the clinical symptoms before death of the sows/gilts that were euthanised or found dead. For sows/gilts with PAD fracture the herd staff observation was correct in 60% of the cases. However, in 43% of the sows/gilts with the PAD arthritis, the herd staff predicted a fracture. This is interesting since it shows that a significant proportion of the sows with arthritis may incorrectly, in the recording of removal reasons, be reported as having fractures. In an earlier study of removal reasons [10], 3.3% of all removed sows (including animals sent to slaughter) had the removal reason "leg fracture". The results from the present study suggest that the incidence of fracture in our earlier study may in fact be lower than reported. This also shows the importance of post mortem examinations to get correct diagnosis. Moreover, knowledge about the aetiology is important for prophylactic measures i.e. for improvements in the herd.

According to the pathological-anatomical findings, including most of the incidental findings, teeth injuries were recorded for 31% of the examined carcasses. This is lower than in two other studies, where 85% had lesions [23] and 42.5% had severe teeth wear [24]. In the present study teeth injuries were often found in the incicives and may have arisen when the sows chewed at steel tubes of the feeding stalls while waiting for the feed. This biting is a common behaviour that often starts when the pigs can hear the feed coming. These results on teeth indicate that teeth injuries may be a problem for sows, influencing their well being and should be further investigated.

## Conclusion

The results from present study show the importance of post mortem examination to obtain the proper diagnoses for sows found dead or being euthanised. The finding that most of the post mortem examined sows had arthritis as main finding needs to be confirmed on a larger number of animals and herds. If this is a general problem, further investigation is needed to find out more about the aetiology so prophylactic measures can be implemented.

## Competing interests

The authors declare that they have no competing interests.

## Authors' contributions

LE participated in the design of the study, evaluated the post mortem findings, performed the descriptive statistics and drafted the manuscript. LES participated in the design of the study, evaluated the post mortem findings and helped to draft the manuscript. NL applied for funding of the project, participated in the design of the study and helped to draft the manuscript. KB participated in the design of the study, evaluated the post mortem findings and helped to draft the manuscript. KA participated in the design of the study and helped to draft the manuscript. AMD participated in the design of the study, evaluated the post mortem findings and contributed significantly to the manuscript. All authors read and approved the final manuscript.
